# Retrospective Comparison of Prehospital Sustained Return of Spontaneous Circulation (ROSC) Rates Within a Single Basic Life Support Jurisdiction Using Manual vs Lund University Cardiac Assist System (LUCAS-2) Mechanical Cardiopulmonary Resuscitation

**DOI:** 10.7759/cureus.26131

**Published:** 2022-06-20

**Authors:** Joshua Mastenbrook, Kathryn E Redinger, Duncan Vos, Cheryl Dickson

**Affiliations:** 1 Emergency Medicine, Western Michigan University Homer Stryker M.D. School of Medicine, Kalamazoo, USA; 2 Epidemiology and Biostatistics, Western Michigan University Homer Stryker M.D. School of Medicine, Kalamazoo, USA; 3 Pediatrics and Adolescent Medicine, Western Michigan University Homer Stryker M.D. School of Medicine, Kalamazoo, USA

**Keywords:** pre-hospital emergency medicine, chest compressions, rosc, cardiopulmonary resuscitation, mechanical cpr, cardiac arrest

## Abstract

Objective

Several studies have examined the impact of mechanical cardiopulmonary resuscitation (CPR) devices among multi-jurisdictional emergency medical services (EMS) systems; however, the variability across such systems can inject bias and confounding variables. We focused our investigation on the effect of introducing the Lund University Cardiac Assist System 2 (LUCAS-2) into a single basic life support (BLS) fire department first response jurisdiction served by a single private advanced life support (ALS) agency, hypothesizing that the implementation of the device would increase prehospital return of spontaneous circulation (ROSC) rates as compared with manual CPR.

Methods

A retrospective observational analysis of adult non-traumatic prehospital cardiac arrest ALS agency records was conducted. Descriptive statistics were computed, and logistic regression was used to assess the impact of CPR method, response time, age, gender, CPR initiator, witnessed status, automated external defibrillator (AED) initiator, and presence of an initial shockable rhythm on ROSC rates. A Chi-square analysis was used to compare ROSC rates among compression modalities both before and after the implementation of LUCAS-2 on July 1, 2011.

Results

From an initial dataset of 857 cardiac arrest records, only 264 (74 pre-LUCAS period, 190 LUCAS-2 period) met inclusion criteria for the primary objective. The ROSC rates were 29.7% (22/74) and 29.5% (56/190), respectively, for manual-only and LUCAS-assisted CPR (p=0.9673). Logistic regression revealed a significant association between ROSC and two of the independent variables: arrest witnessed (OR 3.104; 95% CI 1.896-5.081; p<0.0001) and initial rhythm shockable (OR 2.785; 95% CI 1.492-5.199; p<0.0013).

Conclusions

Analyses support the null hypothesis that there is no difference in prehospital ROSC rates among adult non-traumatic cardiac arrest patients when comparing mechanical-assisted and manual-only CPR. These results are consistent with other larger multi-jurisdictional mechanical CPR studies. Systems with limited personnel might consider augmenting their resuscitations with a mechanical CPR device, although cost and system design should be factored into the decision. Secondary analysis of independent variables suggests that prehospital cardiac arrest patients with a witnessed arrest or an initial rhythm that is shockable have a higher likelihood of attaining ROSC. The power of our primary objective was limited by the sample size. Additionally, we were not able to adequately assess the quality of CPR among the two comparison groups with a lack of consistent end-tidal carbon dioxide (EtCO2) data.

## Introduction

According to the American Heart Association, current evidence suggests that one in every 7.4 people in the United States will suffer from, and many will succumb to, sudden cardiac arrest. The incidence of out-of-hospital cardiac arrest (OHCA) remains alarmingly high, with approximately 356,000 OHCAs annually, yet there is some hope that survival rates of both in-hospital and out-of-hospital cardiac arrests continue to improve over time [1]. This is, in larger part, proposed to be due to decreasing the time to initiating chest compressions and improving the quality of chest compressions during cardiac arrest resuscitation [2]. 

It has been proposed that the use of an automated compression device may improve overall cardiopulmonary resuscitation (CPR) quality because of the ability to achieve consistent compression rate and depth, increase chest compression fraction, minimize rescuer fatigue, and allow for rescuers to complete other tasks [3,4]. The Lund University Cardiac Assist System 2 (LUCAS-2) is one such example, being an automated battery-powered piston-driven mechanical chest compression device (mCPR) with a suction cup at the distal end of the piston. 

There have been several studies published evaluating the effectiveness of mechanically delivered compressions in comparison to manual compressions, yet these have yielded mixed results [5,6,7]. In a 2018 Cochrane review, the authors reviewed 11 clinical studies comparing manual versus mechanical CPR, some citing no difference in outcomes, some with poorer outcomes, and some with improved outcomes [8]. Mechanical chest compressions are not always without harm. Adverse effects of mechanical chest compressions, including pulmonary contusions, lacerations, rib fractures, and pneumothoraces, have been reported and may be a contributing factor as to why we have not witnessed the expected improved survival rates of a mechanical device would predictively provide [3,9]. 

With conflicting data, the American Heart Association 2020 guidelines recommend that manual chest compressions be the foundation of CPR, but mechanical piston-driven devices may be considered in specific settings where high-quality chest compressions may be challenging or dangerous for the provider [10]. 

Here, we present a retrospective observational cohort study evaluating whether prehospital return of spontaneous circulation (ROSC) rates differ across CPR modalities within a single basic life support (BLS) fire department first response jurisdiction. Our hypothesis was that there would be an increase in the rate of sustained ROSC for cases utilizing the LUCAS-2 device.

## Materials and methods

Design and setting 

This was a retrospective observational cohort study in which de-identified prehospital data were obtained from the Life Care Ambulance electronic patient care reporting (ePCR) database in Calhoun County, Michigan, to analyze all non-traumatic out-of-hospital cardiac arrests in which manual or mechanical CPR was performed from July 1, 2011, through October 18, 2017. On July 1, 2011, the Battle Creek Fire Department began utilizing the LUCAS-2 during OHCA resuscitations. The primary objective was to evaluate whether ROSC incidence differs between modalities (manual, LUCAS, LUCAS and manual) during the LUCAS period (July 1, 2011 - October 18, 2017). Secondary objectives included an evaluation of the association between patient characteristics/demographics and achieving prehospital ROSC during the LUCAS period (July 1, 2011 - October 18, 2017) and an evaluation of whether ROSC incidence differs between those given manual CPR in the pre-LUCAS period (January 24, 2008 - July 1, 2011) and those given mechanical CPR during the LUCAS-2 period (July 1, 2011 - October 18, 2017).

The Battle Creek Fire Department is a full-time BLS non-transporting department with seven stations serving a population of 52,350 people spread over 44 square miles. Their emergency medical services (EMS) call volume exceeds 5,000 responses annually. The fire department jurisdiction is served by a single private advanced life support (ALS) ambulance agency, LifeCare Ambulance. This study was approved by the Western Michigan University Homer Stryker MD School of Medicine Institutional Review Board.

Selection of subjects 

Inclusion criteria consisted of any EMS call that included the use of manual CPR, mechanical CPR (LUCAS-2), or both. Additional data elements extracted were time from dispatch to patient contact, who initiated the CPR, witnessed or unwitnessed arrest, who first applied the monitor/defibrillator/automated external defibrillator (AED), initial rhythm shockable or non-shockable, and patient age and gender. Entries missing data pertaining to the parameters listed above, cases of cardiac arrest due to presumed non-cardiac causes as documented in the ePCR (trauma, intoxication, poisoning, etc.), and pediatric cases (age <18) were excluded. 

Objectives and statistical analysis 

The outcome used for primary and secondary objectives is the incidence of return of spontaneous circulation (ROSC), defined as sustained spontaneous circulation until the transfer of care to the medical staff at the receiving hospital. For the primary objective, a Chi-square analysis was used to assess the impact of the introduction of the LUCAS-2 device by comparing the incidence of ROSC achieved in OHCAs utilizing manual chest compressions, the LUCAS device only, or both methods during the LUCAS-2 period from July 1, 2011, through October 18, 2017. 

For the secondary objective, a logistic regression model was utilized to assess the association of independent variables with achieving prehospital ROSC. These independent variables included method (manual, LUCAS-2, both manual and LUCAS-2), time from dispatch to patient contact, individual initiating CPR (lay bystander, medically trained bystander, first responder, EMS personnel), witnessed or unwitnessed status of the arrest, individual first applying the monitor/defib/AED (bystander, first responder/EMS), initial rhythm shockable or non-shockable, and patient age and gender. The unadjusted association for each predictor of sustained ROSC was obtained by running univariate models. Significant predictors among the univariate models were included in a multivariable model to obtain the adjusted association between the predictors and sustained ROSC. 

Lastly, for the secondary analysis comparing sustained ROSC rates for manual CPR in the pre-LUCAS period (January 24, 2008 - July 1, 2011) and LUCAS-2 associated CPR during the LUCAS-2 period (July 1, 2011 - October 18, 2017), a Chi-square analysis was conducted. 

SAS version 9.4 (SAS Institute Inc., Cary, North Carolina) was utilized for analyses. All tests were two-tailed, with significance declared at alpha = 0.05. 

## Results

**Figure 1:**
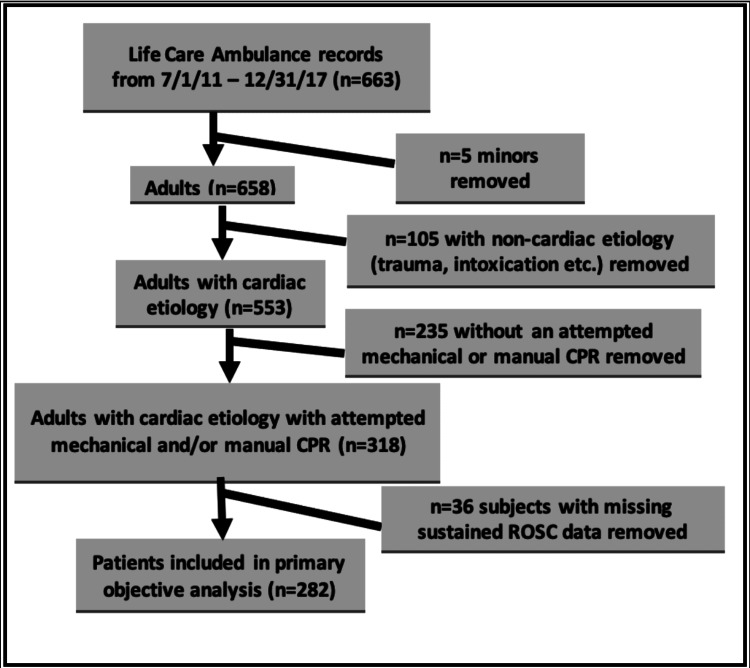
Flow chart of subject inclusion and exclusion CPR - cardiopulmonary resuscitation; ROSC - return of spontaneous circulation

The dataset consisted of n=663 subjects. Excluding those who were less than 18 years of age resulted in n=658 subjects. After excluding any subjects with non-presumed cardiac etiology, 553 subjects remained. After including only subjects in which mechanical or manual CPR was attempted, and excluding 36 subjects because of incomplete data, we had n=282 subjects, which was the final data set to be used for our primary analysis (Figure 1). 

One hundred ten subjects were documented as receiving only manual chest compressions, 80 received only LUCAS-2 compressions, and the remaining 92 received manual and LUCAS-2 compressions during the resuscitation. Patients that received manual only, LUCAS-2 only, and both compression modalities, were similar with respect to age, gender and time from dispatch to patient contact. Furthermore, the frequency with which cases experienced a non-shockable rhythm was similar across the three groups. The frequency with which cases experienced a shockable rhythm was similar across the three groups. In cases where both manual and LUCAS-2 compressions were initiated, there was a greater proportion in which a layperson initiated CPR. Demographic data are shown in Table 1. 

**Table 1:** The distribution of variables of interest for subjects with Manual CPR only, LUCAS only, and both manual and LUCAS. Categorical variables are reported as frequency (percent). Numeric variables (age, and time from dispatch to patient contact) are reported as mean (standard deviation). CPR - cardiopulmonary resuscitation; LUCAS- Lund University Cardiac Assist System; EMS - emergency medical services; PEA - pulseless electrical activity

	Manual CPR only (n=110)	LUCAS only (n=80)	Both manual and LUCAS (n=92)
Arrest			
Witnessed	57 (54.81%)	35 (44.30%)	35 (39.33%)
Unwitnessed	47 (45.19%)	44 (55.70%)	54 (60.67%)
Gender			
Male	69 (62.73%)	46 (58.23%)	54 (58.70%)
Female	41 (32.27%)	33 (41.77%)	38 (41.30%)
First defib by			
Bystander	1 (1.14%)	0	3 (4.05%)
First responder or EMS	87 (98.86%)	64 (100%)	71 (95.95%)
Who initiated CPR			
First responder	33 (30.28%)	26 (32.91%)	23 (25.27%)
Lay person	11 (10.09%)	9 (11.39%)	16 (17.58%)
Lay person family member	13 (11.93%)	8 (10.13%)	10 (10.99%)
Lay person medical provider	20 (18.35%)	7 (8.86%)	19 (20.88%)
Responding EMS personnel	28 (25.69%)	26 (32.91%)	22 (24.18%)
Other	4 (3.67%)	3 (3.80%)	1 (1.10%)
First arrest rhythm			
Ventricular tachycardia, ventricular fibrillation, or unknown shockable	10 (9.80%)	13 (16.25%)	11 (12.09%)
Unknown unshockable, idioventricular/PEA, or asystole	92 (90.20%)	67 (83.75%)	80 (87.91%)
Age (years)	65.81 (15.10)	66.24 (16.62)	65.02 (15.00)
Time from dispatch to patient contact (minutes)	6.37 (3.20)	6.35 (2.42)	6.29 (2.18)

Primary objective: CPR method and sustained ROSC 

There were 110 subjects who underwent manual-only CPR, and of those, 31 (28.18%) attained sustained ROSC. There were 80 subjects who underwent mechanical-only CPR, and of those, 25 (31.25%) attained sustained ROSC. There were also 92 subjects who had both manual and mechanical CPR documented, with 24 (26.09%) achieving sustained ROSC (Table 2). Comparing the incidence of ROSC between these three groups revealed a p-value of 0.7541, indicating no statistically significant difference in the incidence of ROSC between the three groups. Furthermore, when comparing the mCPR-only group and the manual-only CPR group, there was not a significant difference (p=0.6470) in the sustained ROSC rate. 

**Table 2:** Analysis of the sustained ROSC rates for those that received manual CPR only (n=110), those that received LUCAS only (n=80), and those that received both LUCAS and manual (n=92) Analysis shows that during the LUCAS period, there is not a statistically significant difference (p=0.7541) between the three groups. Furthermore, when looking only at the LUCAS-only group and the manual CPR-only group, there is not a significant difference (p=0.6470) in the sustained ROSC rates. CPR - cardiopulmonary resuscitation; LUCAS - Lund University Cardiac Assist System; ROSC - return of spontaneous circulation

Method	Sustained ROSC		
	No	Yes	Total
Both	68 (73.91%)	24 (26.09%)	92
Manual CPR only	79 (71.82%)	31 (28.18%)	110
LUCAS only	55 (68.75%)	25 (31.25%)	80
Total	202	80	282

Secondary objective: demographics and characteristics associated with ROSC 

Logistic regression was used to assess the association between independent variables and sustained ROSC. The variable of a witnessed OHCA was found to have a significant univariate association (p<.0001) with sustained ROSC. Specifically, subjects in which the cardiac arrest was witnessed are 4.1 (95% CI 2.3, 7.3) times more likely to have sustained ROSC than those OHCA cases that were unwitnessed. Additionally, the variable, first arrest rhythm of the patient, was found to have a significant univariate association (p=0.0029) with sustained ROSC. Specifically, subjects in which the first arrest rhythm was ventricular tachycardia, ventricular fibrillation, or unknown shockable rhythm were 3.1 (95% CI 1.5, 6.3) times more likely to have sustained ROSC than are those with non-shockable rhythms. 

Mechanical CPR (p=0.6471), gender (p=0.4009), individual who initiated CPR (p=0.5266), and time from dispatch to patient contact (p=0.9205) were not significant in univariate logistic regression with predicting sustained ROSC. Additionally, using this model, patient age was found to be a significant univariate with a p=0.0405. The average age of those patients with sustained ROSC was 68.5 (95% CI 59.5, 80.5), while the average age of those who did not have sustained ROSC was 64 (95% CI 55, 78). 

The multivariable model yielded moderate prediction power of sustained ROSC (c=0.719). Arrest witnessed, shockable rhythm, and patient age were found to be significant univariate predictors of sustained ROSC. When included in a multivariable model together, shockable rhythm is no longer significant. Cases in which arrests are witnessed have 3.7 times greater odds of achieving sustained ROSC while accounting for shockable rhythm and patient age (Table 3). 

**Table 3:** Secondary analysis of independent variables The table shows the frequency (percent) of sustained ROSC for CPR method, arrest witnessed, shockable rhythm, whom CPR was initiated by, whom the defibrillator was applied by, and patient gender. The median (interquartile) range is reported for patient age and time from dispatch to patient contact. The odds ratio (95% CI) and p-value are reported for each of the univariate logistic regression models.  Variables found to be significant predictors of sustained ROSC in the univariate logistic regression model at alpha=.05 were included in the multivariable logistic regression model. CPR - cardiopulmonary resuscitation; LUCAS - Lund University Cardiac Assist System; EMS - emergency medical services; ROSC - return of spontaneous circulation

	Sustained ROSC achieved		Univariate models		Multivariable model	
	Yes (n=80)	No (n=202)	OR (95% CI)	p-value	OR (95% CI)	p-value
CPR method						
Both manual & LUCAS	24 (26.09%)	68 (73.91%)	0.9 (0.5, 1.7)	p=.7391		
LUCAS only	25 (31.25%)	55 (68.75%)	1.2 (0.6, 2.2)	p=.6471		
Manual only (ref)	31 (28.18%)	79 (71.82%)	-	-	-	-
Arrest witnessed						
Witnessed	54 (42.52%)	73 (57.48%)	4.1 (2.3, 7.3)	p < .0001	3.7 (1.8, 7.4)	p=.0003
Unwitnessed (ref)	22 (15.17%)	123 (84.83%)	-	-	-	-
Shockable rhythm						
Yes	17 (50.00%)	17 (50.00%)	3.1 (1.5, 6.3)	p=.0029	2.4 (0.9, 5.7)	p=.0546
No (ref)	59 (24.69%)	180 (75.31%)	-	-	-	-
CPR initiated by						
First responder or EMS	42 (26.58%)	116 (73.42%)	0.8 (0.5, 1.4)	p=.5266		
Lay individual (ref)	34 (30.09%)	79 (69.61%)	-	-	-	-
Defib. first applied by						
Bystander	0	4 (100.00%)				
First responder or EMS	63 (28.38%)	159 (71.62%)				
Patient gender						
Male	45 (26.63%)	124 (73.37%)	0.8 (.47, 1.35)	p=.4009		
Female (ref)	35 (31.25%)	77 (68.75%)	-	-	-	-
Patient age (years)	68.5 (59.5, 80.5)	64 (55, 78)	1.02 (1.00, 1.04)	p=.0405	1.02 (1.00, 1.05)	p=.0479
Time from dispatch to patient contact (minutes)	6.2 (4.6, 8.1)	6.0 (4.4, 8.2)	1.0 (0.9, 1.1)	p=.9205		

## Discussion

Out-of-hospital cardiac arrest scenes may be chaotic and contain many variables that combine to determine the likelihood of survival. It has been well demonstrated that good quality CPR is a major contributor to better overall survival rates. It has also been suggested that mechanical CPR devices may be able to provide superior chest compressions and that their resulting ability to improve vascular pressures and, in turn, coronary and cerebral perfusion may portend a survival benefit when used in the prehospital setting [2,11].

Data presented herein are similar to national cardiac arrest data from the CARES network for 2013-2017 [12], which reports 61.4% male, 50.7% unwitnessed arrest, 80.4% with unknown unshockable, idioventricular/pulseless electrical activity (PEA), or asystole, median age 64 years, and sustained ROSC rate of 32.3%. We observed 87.5% with first arrest rhythm of unknown unshockable, idioventricular/PEA, or asystole, which compares nationally to CARES data from 2013-2017 reporting 81.4% of similar first arrest rhythm. Although our study was underpowered to detect differences based on gender, the observed distribution of gender (60.14% male) is similar to national data from the CARES registry from 2013 to 2017 of 61.4% male. 

In our study, there was not sufficient evidence to declare a difference in ROSC rates among adult non-traumatic OHCA patients when comparing mechanical-assisted and manual-only CPR. With a greater sample size in a future study, there may be sufficient power to determine equivalence or non-inferiority. As a single system study, the sample size was relatively small; however, these results are consistent with other larger multi-jurisdictional mechanical CPR studies [13]. The mechanical CPR device did not produce superior ROSC rates as compared to cases using manual compressions and does require significant capital investment and system design considerations. Therefore, when deciding whether to introduce a mechanical chest compression device into an EMS system, the benefit of the mechanical CPR device might not be on the survival advantage to the patient but in that it allows the team to focus their attention on other aspects of the resuscitation, such as airway management, vascular access and addressing reversible causes of the arrest, especially in systems with limited personnel. 

In our secondary objective analysis of out-of-hospital cardiac arrests during the LUCAS period, patients with a witnessed arrest or an initial shockable rhythm had a higher likelihood of sustained ROSC. This is consistent with the literature in that survival from out-of-hospital cardiac arrest is more likely among those with witnessed arrests and those found in ventricular tachycardia or ventricular fibrillation [14]. As one cannot alter the presenting rhythm of the cardiac arrest patient, EMS systems looking to improve survival of out-of-hospital cardiac arrest should focus their efforts and investments on timely delivery of interventions. 

Limitations 

As a retrospective observational study, we were not able to randomize subjects and noted there was a lack of consistently reported end-tidal carbon dioxide data that could have served as a proxy for CPR quality. This study was also subject to selection bias, as the decision to utilize the mechanical CPR device was left to the first responders. Additionally, our primary endpoint focused on prehospital sustained ROSC rather than hospital discharge cerebral performance category scores; therefore, inferences into long-term (good neurological) survival are lacking. 

We observed a small clinical difference in sustained ROSC rate between manual CPR (28.18%) and LUCAS-2 CPR (31.25%); however, our study was not sufficiently powered to detect this difference. Post-hoc power analysis indicated that to truly determine whether these two rates differ with a power of 0.8, a sample size of n=3,542 cases per group would be required. Non-inferiority could be determined with a 5% equivalence margin given a total sample size of n=2894. 

Additionally, documentation of the utilization of the LUCAS-2 and/or manual-only CPR may be inconsistent. The documented frequency of manual CPR administration was n=110 or 39% of our included cases; however, it is unclear whether manual-only compressions were administered for these patients or if LUCAS-2 compressions were also administered and not documented. Nonetheless, the secondary analysis revealed that there was not a significant difference detected (p=.9673) in the sustained ROSC rate before and after the LUCAS-2 device was introduced into this EMS system on July 1, 2011. 

## Conclusions

In this single agency small study, the use of the LUCAS-2 mechanical CPR device was not associated with better or worse sustained ROSC rates when compared to manual chest compressions. The benefit of using a mechanical CPR device may be more related to the reduction of the cognitive and physical workload of the team, allowing devoted attention to other aspects of the resuscitation. The presence of a witnessed arrest or the first rhythm being identified as shockable appears to afford a short-term survival benefit with improved chances of achieving sustained ROSC. 
